# BAR-UNet: a boundary-aware and appearance-robust framework for polyp segmentation

**DOI:** 10.3389/fonc.2026.1873111

**Published:** 2026-07-22

**Authors:** Yiliu Xu, Lingling Liu, Meiwen Tang

**Affiliations:** 1 Guangxi University of Chinese Medicine, Nanning, China; 2 Hubei University of Chinese Medicine, Wuhan, China

**Keywords:** boundary-aware segmentation, colonoscopy, colorectal polyps, consistency regularization, polyp segmentation

## Abstract

**Background:**

Colorectal cancer is a leading cause of cancer-related mortality, and reliable polyp segmentation during colonoscopy is critical for early intervention. Existing deep learning segmentors often produce blurred boundaries and are sensitive to appearance variation across endoscopy devices.

**Methods:**

We propose BAR-UNet, a ResNet-34 encoder–decoder with a boundary-aware head and mask-guided appearance consistency learning (MACL). On the public Kvasir-SEG dataset we compare BAR-UNet with classical semantic segmentors (FCN-ResNet50, DeepLabV3-ResNet50), detection-based segmentors (YOLOv8-Seg, YOLOv11-Seg, Mask R-CNN), polyp-specific CNN baselines, and modern enhanced segmentors (DeepLabV3+, SegFormer-B2, MedSAM, SAM-Med2D) under a unified 70/10/20 split, multi-seed evaluation protocol.

**Results:**

BAR-UNet achieves Dice 0.8798 ±0.0024 and IoU 0.7913 ± 0.0029 on Kvasir-SEG, outperforming all compared methods. Zero-shot evaluation on CVC-ClinicDB, CVC-ColonDB, and ETIS-Larib yields Dice/IoU of 0.831/0.748, 0.776/0.682, and 0.728/0.631, respectively. Ablation studies, boundary metrics (F_1_^^bd^, MAE, HD95), paired Wilcoxon tests, and MACL sensitivity analyses confirm that the boundary head and MACL are complementary.

**Discussion:**

BAR-UNet improves boundary precision and appearance robustness with modest computational overhead on an NVIDIA RTX 4060 GPU. The method is a promising research prototype for computer-aided polyp analysis; prospective clinical validation is required before deployment.

## 1 Introduction

Colorectal cancer (CRC) is one of the leading causes of cancer morbidity and mortality worldwide, and timely detection and removal of precancerous polyps during colonoscopy is critical for improving patient outcomes Winawer et al. (1) Zauber et al. (2). Over the past decade, a large body of computeraided diagnosis (CAD) work has investigated automatic polyp detection and segmentation in colonoscopy videos Taha et al. (3) Tajbakhsh et al. (4)Lee et al. (5) Wang et al. (6) Jha et al. (7)Wu et al. (8). In recent years, deep learning has substantially advanced automated polyp analysis, especially on publicly available benchmarks such as Kvasir-SEG Jha et al. (9); bibliometric and systematic reviews document rapid integration of neural networks into biomedical AI pipelines Chen et al. (10) Zhang et al. (11), while data-intensive molecular studies in other clinical domains similarly illustrate the expanding role of learning-based biomedical analysis Zhang et al. (12). Compared with mere bounding-box detection, *pixel-wise* polyp segmentation provides more precise localization of lesion boundaries, which is important for assessing polyp size, morphology, and resection margins. However, robust polyp segmentation in real clinical practice remains challenging due to strong variations in appearance: different patients, different endoscopy devices, diverse bowel preparation quality, and fluctuating illumination conditions all lead to significant domain shifts and noisy boundaries.

A large body of work has explored both classic semantic segmentation networks and detection-based instance segmentation for polyp analysis. U-Net and its variants Ronneberger et al. (13)Zhou et al. (14) remain strong baselines in medical image segmentation due to their encoder–decoder architecture and skip connections. Fully convolutional networks (FCN) Long et al. (15) and DeepLabV3/V3+ Chen et al. (16), 17 introduce multi-scale context aggregation and dilated convolutions, and have been successfully applied to endoscopic imagery, alongside broader medical imaging segmentation studies based on convolutional features or partial differential equation (PDE) regularization Yao et al. (18) Tian and Zhang (19) Tian and Chen (20) Bin-Salem et al. (21). On the other hand, two-stage detectors such as Mask R-CNN He et al. (22) and one-stage models from the YOLO family Redmon et al. (23) Jocher et al. (, 24), 25, as well as improved Faster R-CNN and small-target detectors for challenging scenes Yin and Chen (26) Liu et al. (27); liang Zhang et al. (28) provide unified detection and instance-level segmentation pipelines. Despite their progress, existing methods still suffer from a key limitation in the polyp scenario: they often produce uncertain and fragmented boundaries when the polyp appearance changes across devices and imaging conditions, leading to degraded Dice/IoU scores especially on cross-domain or low-quality frames.

This limitation can be further decomposed into two tightly related challenges. First, boundary-awareness is insufficient: many encoder–decoder or detection-style segmentors optimize primarily for region overlap, so the network tends to focus on the central polyp area while under-emphasizing fine boundary structures; this makes the predictions particularly vulnerable near specular highlights, motion blur, and low-contrast edges. Second, appearance robustness is weak: current models are usually trained with standard augmentations and a single supervised mask loss, which does not explicitly encourage consistency under strong appearance changes such as device style, illumination shifts, or varying bowel cleanliness. As a result, the same polyp region may yield noticeably different masks when the visual appearance changes, even though the underlying anatomy is identical. These two challenges are amplified in gastrointestinal endoscopy, where boundary cues are subtle and domain shifts between patients and devices are ubiquitous.

To address the above challenges, we propose a boundary-aware and appearance-robust polyp segmentation framework, termed BAR-UNet (Boundary-Aware and appearance-Robust U-Net), built upon a ResNetbased encoder–decoder backbone. Concretely, we introduce two complementary innovations. (1) A dedicated boundary head that learns an explicit polyp boundary map from intermediate decoder features, coupled with a boundary supervision signal derived from the ground-truth mask; this guides the network to pay more attention to thin and low-contrast edges, alleviating the under-segmentation of boundary regions. (2) A mask-guided appearance consistency learning (MACL) strategy, where strongly augmented views of the same image are fed through the network and a consistency loss is enforced on the predicted masks within a mask-dilated neighborhood; this explicitly encourages the model to produce stable masks under large appearance perturbations caused by different devices and imaging conditions. Together, these two components enhance both boundary precision and cross-appearance robustness.

The main contributions of this work are summarized as follows:

- We present a comprehensive study of polyp segmentation on the Kvasir-SEG dataset, comparing detection-based instance segmentation (YOLO-Seg, Mask R-CNN) with classic semantic segmentation baselines (FCN, DeepLab, U-Net family) under a unified evaluation protocol with Dice and IoU metrics.
- We identify and analyze two key challenges for robust polyp segmentation in realistic endoscopy— insufficient boundary-awareness and weak appearance robustness across devices and imaging conditions—and formulate them as concrete design targets for model development.
- We propose the BAR-UNet framework with a boundary-aware head and a mask-guided appearance consistency learning (MACL) scheme, which jointly improve boundary quality and robustness to strong appearance variations.
- We evaluate cross-dataset generalization on CVC-ClinicDB, CVC-ColonDB, and ETIS-Larib, and report boundary-specific metrics to substantiate the boundary-aware design.

## 2 Related work

In this section, we review prior work on polyp segmentation and detection, with emphasis on boundary quality and robustness to appearance variation. We then explain how the remaining gaps motivate our BAR-UNet design.

### 2.1 Polyp segmentation with semantic segmentation networks

Early deep-learning-based polyp segmentation methods largely adopt generic semantic segmentation architectures. Jha et al. Jha et al. (9) introduced the Kvasir-SEG dataset and benchmarked several fully convolutional architectures, including FCN Long et al. (15) and U-Net Ronneberger et al. (13), demonstrating that encoder–decoder models with skip connections are effective baselines for medical image segmentation. Ronneberger et al. (13) proposed U-Net, which has become the de facto standard architecture in biomedical segmentation due to its symmetric contracting and expanding paths and rich feature fusion. Zhou et al. Zhou et al. (14) further extended this line of work with UNet++, introducing nested and dense skip connections to better bridge semantic gaps between encoder and decoder. These architectures generally offer high Dice/IoU on in-distribution test sets, and are relatively lightweight and easy to train.

More recent approaches specifically target polyp segmentation with customized backbones and loss functions. Jha et al. (29, 30) proposed ResUNet++ and conducted a comprehensive study of U-Net variants for colonoscopy images, introducing residual blocks, squeeze-and-excitation modules, and attention mechanisms to improve representation capacity. Fan et al. Fan et al. (31) presented PraNet, which leverages reverse attention and edge guidance to highlight polyp regions and refine boundaries, achieving state-of-the-art performance on multiple polyp datasets. Huang et al. Huang et al. (32) designed HarDNet-MSEG, a lightweight yet accurate model based on harmonic dense connections, emphasizing the trade-off between accuracy and efficiency. FLDNet Wei and Zhou (33) further exploits long-distance dependencies via a pyramid vision transformer encoder for foreground-aware polyp segmentation. Plug-in geometric priors have also been explored: GPM Vazquez et al. (34) injects depth-based structural cues into U-Net skip connections and reports consistent gains across five public benchmarks, though it requires an additional depth-estimation stage. Other works explore integrating geometric constraints and richer contextual cues Guo et al. (35) or unifying detection and segmentation within single deep models Jha et al. (7)Wu et al. (8). These methods illustrate that incorporating attention, boundary cues, and carefully designed decoders can significantly enhance segmentation quality.

Despite these advances, many polyp segmentors are still trained and evaluated under relatively fixed imaging conditions and can degrade on data from other endoscopy devices or acquisition settings. In addition, region-overlap losses combined with conventional augmentations often place limited emphasis on thin boundary pixels—a concern shared by cross-domain edge detection pipelines Wang et al. (36)—so masks may become blurred or unstable when illumination, bowel preparation, or device appearance changes substantially; analogous robustness challenges arise in deep anomaly screening under domain shift Zhang et al. (37).

More recently, foundation models for medical image segmentation have emerged as strong, architectureagnostic baselines. Segment Anything (SAM) Kirillov et al. (38) and its medical adaptations— MedSAM Ma et al. (39) and SAM-Med2D Cheng et al. (40)—leverage large-scale pretraining and promptable decoders, while enhanced dilated CNN and transformer decoders such as DeepLabV3+ Chen et al. (17) and SegFormer Xie et al. (41) remain widely used fully automatic segmentors. Concurrent polyp-specific designs continue to advance attention and semantic priors, including AGAM/AAPCNet Khan et al. (42), ADPNet Khan et al. (43), LMSGNet Wang et al. (44), and SFGNet Wang et al. (45) (**Table 1**); related lines of work also explore geometric priors in reconstruction Wang et al. (46), attention-enhanced image analysis Li and Dong (47), and multi-view feature fusion Qian et al. (48) Yu et al. (49, 50). Graph-based deep models have further been applied to clinical motion assessment Zhang et al. (51). These methods achieve strong scores on standard benchmarks such as Kvasir-SEG, CVCClinicDB, CVC-ColonDB, and ETIS-Larib, yet they generally improve accuracy by richer pretraining, prompt engineering, or decoder capacity rather than by enforcing mask stability under strong within-image appearance perturbations.

**Table 1:** Comparison of design emphasis in representative recent polyp segmentors and BAR-UNet.

Method	Main design focus	Boundary modeling	Appearance robustness	External prior
AGAM	Multiscale attention (Res2Net)	Decoder attention	Standard augmentation	None
ADPNet	CNN + Transformer dual path	Weighted IoU/focal loss	Standard augmentation	None
LMSGNet	CLIP semantics + edge guidance	SEGB/MSEF	Cross-dataset training	CLIP
SFGNet Wang et al. (45)	Semantic + frequency (COD)	Structure/frequency cues	COD benchmarks	None
BAR-UNet	Boundary head + MACL	Explicit boundary loss	MACL consistency	None

**Table 1** contrasts how representative recent methods and BAR-UNet address boundaries and appearance variation. Our design follows a different but complementary route: a lightweight ResNet-34 U-Net with an explicit boundary prediction branch supervised by morphological edge maps, and mask-guided appearance consistency learning (MACL) that encourages agreement between strongly augmented views within a mask-dilated neighborhood around the lesion. Quantitative comparison with literature-reported results on overlapping datasets is provided in Section 4.3.

### 2.2 Detection-based and YOLO-style polyp analysis

In parallel, a number of works investigate detection-based frameworks for colorectal polyp analysis. Lee et al. Lee et al. (5) employed a deep convolutional neural network for real-time polyp detection during colonoscopy, demonstrating that detection accuracy and speed can be sufficient for clinical assistance; related CNN-based spatial localization frameworks Wang et al. (52) further illustrate the broader role of convolutional detectors in vision tasks. Wang et al. Wang et al. (6) developed an automatic polyp detection system evaluated in multicenter clinical trials, showing that AI-assisted colonoscopy can reduce adenoma miss rates. These approaches, however, typically output bounding boxes rather than pixel-wise masks; hence, they are well suited for lesion localization and counting, but less informative for tasks requiring precise boundary delineation such as measuring resection margins or performing volume estimation.

More recently, instance segmentation models have been explored for polyp analysis. Mask R-CNN He et al. (22) has been adopted to jointly localize and segment polyps at the instance level. Lewis et al. Lewis et al. (53) proposed a dual encoder–decoder network (PSNet) for polyp segmentation, while Akbari et al. Akbari et al. (54) developed an FCN-based segmentation method with patch selection and post-processing. However, two-stage detectors such as Mask R-CNN are often computationally demanding, and their performance can still be sensitive to imaging artifacts and domain shifts across endoscopy devices.

One-stage detectors and segmentation heads from the YOLO family have also gained popularity due to their efficiency. Redmon et al. Redmon et al. (23) introduced the original YOLO framework, and subsequent versions such as YOLOv5 and YOLOv8 Jocher et al. (24, 25) have been extended with segmentation heads that simultaneously predict bounding boxes, class labels, and masks. Prior studies Jha et al. (7) Wu et al. (8) others (55) explored YOLO-style and general detection–tracking pipelines for polyp or related vision tasks, achieving high precision and recall with real-time throughput. Optimized deep detectors have also been studied for low-contrast or small targets Gao et al. (56) Gayal and Patil (57) Lei et al. (58). Because polyps are often small relative to the endoscopic field of view, recent transformer-based small-object detectors have further studied background query noise and efficient query retrieval Zeng et al. (59); nevertheless, such detection-centric designs still prioritize bounding-box or query-level outputs rather than dense mask delineation. While these methods naturally fit into a detectioncentric evaluation with mAP metrics and can provide instance-wise masks, their segmentation heads are usually shallow and primarily optimized for detection objectives. Consequently, they may underutilize fine-grained boundary information and lack explicit mechanisms to enforce mask stability under strong appearance perturbations.

### 2.3 Broader context from adjacent AI and engineering research

Medical image segmentation increasingly intersects with general deep learning, intelligent systems, and edge computing. Rather than treating these areas as direct competitors to polyp segmentors, we briefly relate them to boundary-aware and deployment-aware CAD design; the following citations are illustrative connections rather than an exhaustive survey.

Representation learning and adaptive training. Sample-efficient learning has been explored through active learning Li et al. (60), heterogeneous graph embedding Wang et al. (61) Zhou and Abbas (62), and temporal network modeling Abbas et al. (63) Fan et al. (64). Reinforcement-learning policy regulation Zhao et al. (65) and multi-source temporal fusion Fan et al. (64) further illustrate how hybrid CNN–transformer architectures address non-stationary signals—a setting analogous to appearance shifts across endoscopy sessions.

Vision-centric deep pipelines. Multistep feature extraction and stacked ensemble classifiers support security-sensitive image analysis Naeem et al. (66–68); Dong et al. (69). Related deep pipelines have also been used for publication-impact forecasting Bento et al. (70), highlighting how convolutional representations transfer across application domains.

Channel coding and synchronization. Quasi-cyclic LDPC code design Xu et al. (71, 72); Zhou et al. (73) Xu et al. (74) Zhu et al. (75) Xu et al. (76) Wang et al. (77) and reverse synchronous transmission Zhang et al. (78) underpin reliable low-latency data links. Complementary studies on relay energy efficiency Zhu et al. (79) Xu et al. (80), fractal circuit modeling Tian et al. (81), and array-signal synchronization Wang (82, 83) are relevant when colonoscopy AI must coexist with bandwidth-constrained clinical networks.

Wireless throughput, localization, and harmonic analysis. Throughput optimization in wirelesspowered NOMA and backscatter systems Zhou (84), global residue harmonic balance Chen and Liu (85), and dual-mode radar localization Fang et al. (86) exemplify signal-processing tools that complement edge deployments of medical vision systems.

Network security, IoT analytics, and edge offloading. Surveys on DDoS attacks Dong et al. (87), active-learning traffic classification Dong (88), software-defined-network intrusion detection Dong and Sarem (89), and authentication in intelligent transportation Dong et al. (90) underscore security requirements for connected clinical devices. Sybil-resilient localization Dong et al. (91), vehicular fog transmission Zhang and Li (92), quantum-swarm task offloading Dong et al. (93), game-theoretic IoT offloading strategies Ding and Zhang (94), and improved PBFT consensus Dong et al. (95) further motivate privacy-preserving federated designs Zhu and Niu (96).

Energy systems, smart cities, and operational learning. Virtual energy-hub optimization Chen and Zhang (97) Wang et al. (98) Yuan et al. (99) Zhang et al. (100), EV-aware smart grids Jiang et al. (101), urban mobility learning Jiang et al. (102), sand-cat path planning Wang et al. (103), warehouse task planning Zhang et al. (104), and bionic-robot simulation Zhang (105) show how learning algorithms are deployed under real-world resource constraints beyond the endoscopy suite.

Materials-aware and biomedical-adjacent sensing. Fiber and photonic sensors Wang et al. (106, 107); Jiang et al. (108)Meng et al. (109, 110) together with bio-responsive hydrogels and CO_2_capture materials Luo et al. (111)Chen et al. (112)Pan et al. (113) reflect cross-disciplinary sensing research in instrument-heavy clinical environments.

In summary, existing semantic segmentation and detection/instance-segmentation approaches have significantly advanced automated polyp analysis, yet most of them do not explicitly and jointly address (i) boundary-aware segmentation under challenging imaging conditions, and (ii) robustness of the predicted masks across substantial appearance changes induced by different devices, illumination patterns, and bowel preparation quality. Motivated by these gaps, we propose BAR-UNet, a boundary-aware and appearanceconsistent polyp segmentation framework that integrates a dedicated boundary head and a mask-guided appearance consistency learning (MACL) strategy, as detailed in the next section.

## 3 Methods

**Figure 1:**
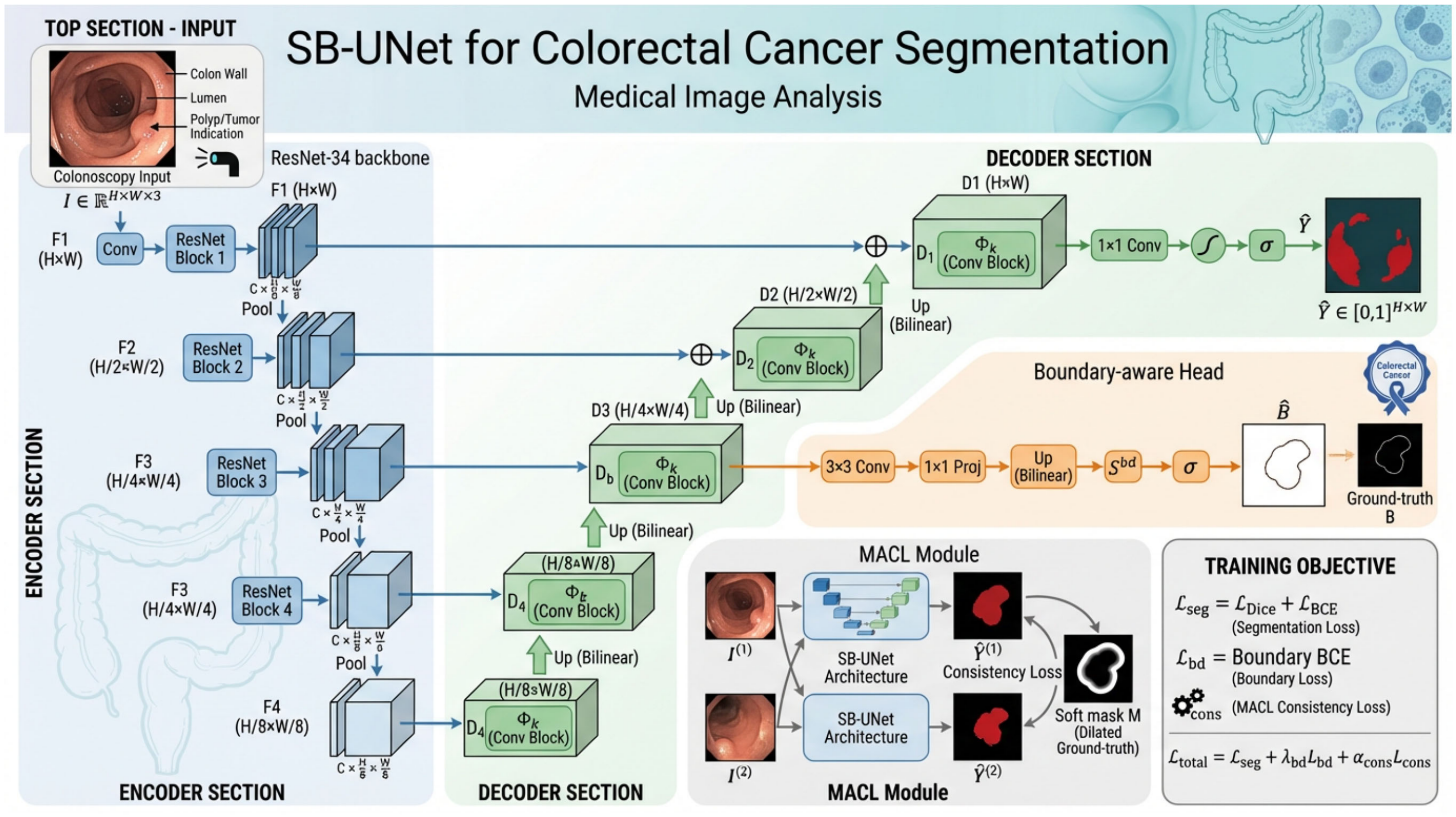
Overall architecture of the proposed BAR-UNet. The model adopts a ResNet-34 encoder and a U-Net-style decoder with skip connections. A boundary-aware head is attached to the decoder features to explicitly learn polyp boundaries, while a mask-guided appearance consistency learning (MACL) module enforces prediction consistency between strongly augmented views within a mask-dilated neighborhood around the polyp.

In this section, we present the proposed BAR-UNet for polyp segmentation. We first describe the overall encoder–decoder architecture, then detail the boundary-aware head and the mask-guided appearance consistency learning (MACL) module, and finally summarize the overall training objective. The overall architecture of the proposed BAR-UNet, including the boundary-aware head and mask-guided appearance consistency learning (MACL) module, is illustrated in **Figure 1**. The mathematical formulations of the encoder–decoder architecture, boundary and consistency losses, evaluation metrics, and complexity analysis are given in Equations (1)–(13).

### 3.1 Overall architecture of BAR-UNet

Our BAR-UNet follows a U-Net-style encoder–decoder design with skip connections, instantiated with a ResNet backbone. Let $I\in {\mathbb{R}}^{H\times W\times 3}$ denote the input colonoscopy image. A ResNet-34 encoder extracts a hierarchy of feature maps {*F*_1_*,F*_2_*,F*_3_*,F*_4_} at progressively lower spatial resolutions and higher semantic levels. The decoder then reconstructs a high-resolution feature representation by a sequence of up-sampling and fusion operations:

(1) $$D_{k}={\text{Φ}}_{k}\left(\text{Up}\left(D_{k+1}\right)⊕F_{k}\right),\;k=4,3,2,1,$$

where *D_k_* denotes the decoder feature at level *k*, Up(·) is bilinear up-sampling, ⊕ denotes channel-wise concatenation, and Φ*_k_*(·) is a small convolutional block. The final segmentation logits map $S\in {\mathbb{R}}^{HesW}$ is obtained by applying a 1 × 1 convolution and up-sampling to *D*_1_:

(2) $$S=\text{Ψ}\left(D_{1}\right),\;\;\hat{Y}=\sigma \left(S\right),$$

where Ψ(·) denotes the segmentation head and *σ*(·) is the sigmoid function producing a foreground probability map $\hat{Y}\in {\left[0,1\right]}^{H\times W}$.

### 3.2 Boundary-aware head and loss

Colonoscopic polyps often have thin and low-contrast boundaries. To explicitly emphasize these structures, we attach a boundary-aware head to an intermediate decoder feature map *D_b_*(e.g., at 1*/*4 resolution), motivated by the importance of explicit edge cues in low-contrast segmentation Wang et al. (36). Given the ground-truth binary mask *Y* ∈ {0,1}*^H^*^×^*^W^*, we derive a boundary ground-truth map *B* ∈ {0,1}*^H^*^×^*^W^* by applying a morphological gradient operatorwith a 3×3 structuring element: *B* = dilate(*Y*,3)−erode(*Y*,3), yielding a one-pixel-wide boundary band unless otherwise noted.

The boundary head consists of a 3 × 3 convolution followed by a 1 × 1 projection and up-sampling to the input resolution, yielding boundary logits *S*^bd^ and probabilities $\hat{B}=\sigma \left(S^{\text{bd}}\right)$. We supervise $\hat{B}$ with a binary cross-entropy (BCE) loss focused on boundary pixels:

(3) $${ℒ}_{\text{bd}}=-\frac{1}{\left|\Omega \right|}\sum_{p\in \text{Ω}}\left[B_{p}\log\;{\hat{B}}_{p}+\left(1-B_{p}\right)\log\;(1-{\hat{B}}_{p})\right],$$

where Ω indexes spatial locations. This loss encourages the decoder to preserve fine boundary details that might otherwise be smoothed out by the region loss.

### 3.3 Mask-guided appearance consistency learning

Endoscopic images exhibit large appearance variations across devices and acquisition conditions. To enhance robustness, we introduce a mask-guided appearance consistency learning (MACL) module. For each training sample, we generate two strongly augmented views *I*^(1)^ and *I*^(2)^ of the same image using color jitter, blur, and other appearance transformations that do not alter the underlying anatomy. These two views are passed through the shared BAR-UNet to produce prediction maps ${\hat{Y}}^{\left(1\right)}$ and ${\hat{Y}}^{\left(2\right)}$.

Instead of enforcing consistency everywhere, which may over-constrain uncertain background regions, we restrict the consistency loss to a mask-dilated neighborhood around the polyp. Let *M* denote a soft mask obtained by dilating the ground-truth *Y* with a disk structuring element of radius *r* = 5 pixels (after resizing to 384 × 384) and clipping to [0,1]. We define the MACL loss as

(4) $${ℒ}_{\text{cons}}=\frac{1}{\left|\Omega \right|}\sum_{p\in \text{Ω}}M_{p}{\left\|{\hat{Y}}_{p}^{\left(1\right)}-{\hat{Y}}_{p}^{\left(2\right)}\right\|}_{2}^{2},$$

where only locations with *M_p_* > 0 (around the polyp region) contribute. This encourages the network to produce stable masks under strong appearance perturbations specifically in anatomically relevant regions.

### 3.4 Overall training objective

The primary segmentation objective combines a Dice loss and a binary cross-entropy (BCE) loss between the predicted mask $\hat{Y}$ and the ground-truth *Y*:

(5) $${ℒ}_{\text{seg}}={ℒ}_{\text{Dice}}\left(\hat{Y},Y\right)+{ℒ}_{\text{BCE}}\left(\hat{Y},Y\right),$$

where

(6) $${ℒ}_{\text{Dice}}\left(\hat{Y},Y\right)=1-\frac{2\sum_{p}{\hat{Y}}_{p}Y_{p}}{\sum_{p}{\hat{Y}}_{p}+\sum_{p}Y_{p}+\epsilon }$$

with a small constant *ϵ* for numerical stability, and L_BCE_ is the standard pixel-wise BCE.

The full training objective for BAR-UNet integrates the segmentation, boundary, and consistency terms:

(7) $${ℒ}_{\text{total}}={ℒ}_{\text{seg}}+\lambda_{\text{bd}}\;{ℒ}_{\text{bd}}+\alpha_{\text{cons}}\;{ℒ}_{\text{cons}},$$

### 3.5 Discussion and time complexity of the proposed design

The two main innovations of BAR-UNet—the boundary-aware head and the mask-guided appearance consistency learning (MACL) module—are designed to explicitly address the two challenges identified in the Introduction: insufficient boundary-awareness and weak appearance robustness. The boundary head introduces an auxiliary prediction branch supervised by a boundary map derived from the ground-truth mask. This additional supervision encourages the decoder to preserve fine edge details, reducing the typical under-segmentation of thin or low-contrast polyp boundaries observed in standard U-Net-style models. MACL, on the other hand, enforces prediction consistency between strongly augmented views of the same lesion within a mask-dilated neighborhood, thereby stabilizing the segmentation under substantial appearance variations caused by different devices, illumination patterns, and bowel preparation conditions.

From a computational perspective, both components are lightweight compared with the backbone encoder– decoder. Let $T_{\text{UNet}}\left(H,W\right)$ denote the complexity of a vanilla ResNet-based U-Net at input resolution *H* × *W*. The boundary head operates on an intermediate feature map at spatial resolution $\left(H^{\prime }W^{\prime }\right)$ with $H^{\prime }W^{\prime }\ll H,W$ (e.g., 1*/*4 resolution), and consists of only a few convolutions, so its additional cost can be written as $O\left(H^{\prime }W^{\prime }k_{\text{bd}}^{2}C_{\text{bd}}^{2}\right)$, which is small relative to $T_{\text{UNet}}\left(H,W\right)$. The MACL branch requires processing two strongly augmented views through the shared network and computing a mask-weighted consistency loss. In practice, the two views are processed within the same training step and share all weights, and the consistency loss is evaluated only within a narrow mask-dilated band around the polyp. As a result, the effective complexity of BAR-UNet can be summarized as

(8) $$\begin{array}{ll} T_{\text{BAR}-\text{UNet}}\left(H,W\right)\approx \; & T_{\text{UNet}}\left(H,W\right)\\ +O\left(H^{\prime }W^{\prime }k_{\text{bd}}^{2}C_{\text{bd}}^{2}\right)+O\left(HWC_{\text{seg}}\right) \end{array}$$

which preserves the same big- O time complexity as a conventional U-Net with comparable depth.

Compared with detection-based models such as YOLO-Seg and Mask R-CNN, which operate at higher input resolutions (e.g., 640 × 640) and include additional detection heads for bounding box regression and classification, BAR-UNet focuses solely on dense pixel-wise prediction at 384 × 384 resolution. This leads to a more favorable accuracy–efficiency trade-off for pure segmentation tasks: BAR-UNet achieves higher Dice and IoU than the considered detection-based baselines (**Table 2**), while maintaining a moderate number of parameters and FLOPs, as quantified in the empirical complexity analysis in Section 4. Overall, the proposed architectural modifications add only modest computational overhead to a standard U-Net backbone, yet bring clear gains in boundary quality and appearance robustness.

**Table 2:** Overall segmentation performance on the Kvasir-SEG test set.

Method	Dice ↑	IoU ↑
FCN-ResNet50	1,0,0 0.8688 ± 0.0031	1,0,0 0.7761 ± 0.0038
DeepLabV3-ResNet50	1,0,0 0.8513 ± 0.0042	1,0,0 0.7540 ± 0.0045
Mask R-CNN	1,0,0 0.7646 ± 0.0068	1,0,0 0.6621 ± 0.0072
YOLOv8-Seg	1,0,0 0.8568 ± 0.0035	1,0,0 0.7830 ± 0.0040
YOLOv11-Seg	1,0,0 0.8304 ± 0.0041	1,0,0 0.7543 ± 0.0048
Ours	1,0,0 0.8798 ± 0.0024	1,0,0 0.7913 ± 0.0029

We report pixel-level Dice and IoU (mean ± std over three random seeds: 42, 123, 456). “Ours” denotes BAR-UNet with both boundary-aware head and MACL. Best results are in bold.

## 4 Experiments

### 4.1 Experimental setup

#### 4.1.1 Dataset and splits

We conduct experiments on the publicly available Kvasir-SEG dataset Jha et al. (9), which contains 1,000 high-resolution colonoscopy still images with corresponding pixel-wise polyp annotations and has been widely adopted in recent polyp segmentation studies Jha et al. (29, 30); Fan et al. (31) Huang et al. (32)Wang et al. (44). Kvasir-SEG is distributed as independent annotated frames rather than explicit video sequences; we therefore perform a random image-level split and verify that no filename duplication occurs across partitions. The dataset is partitioned into non-overlapping training, validation, and test subsets with a fixed ratio of 70%/10%/20%, corresponding to 700, 100, and 200 images, respectively.

To assess generalization beyond Kvasir-SEG, we additionally evaluate models trained on Kvasir-SEG without fine-tuning on three open-access external polyp benchmarks: CVC-ClinicDB (612 images), CVCColonDB (380 images), and ETIS-Larib (196 images). These datasets, together with Kvasir-SEG, are widely used open-source colonoscopy segmentation benchmarks; no private or proprietary clinical data were used in this study. All external images are resized to 384×384 and evaluated with the same binarization threshold and metric script used for Kvasir-SEG.

Following common practice, we resize all images and masks to a fixed resolution of 384 × 384 for the BAR-UNet-based methods and classical semantic segmentation baselines. For YOLO-based segmentation models, we follow the official Ultralytics default input size of 640 × 640 in the main comparison, and additionally report a resolution-matched variant at 384 × 384 in **Table 3** to isolate the effect of input resolution.

**Table 3:** Effect of input resolution on segmentation performance (Kvasir-SEG test set, seed 42).

Method	Input size	Dice	IoU
YOLOv8-Seg	640 × 640	0.8568	0.7830
YOLOv8-Seg	384 × 384	0.8491	0.7742
YOLOv11-Seg	640 × 640	0.8304	0.7543
YOLOv11-Seg	384 × 384	0.8246	0.7481
Ours (BAR-UNet)	384 × 384	0.8774	0.7886

All methods are trained on the same training split and evaluated on the same validation and test splits to ensure a fair comparison. We fix the random seed (seed = 42 for the main run; additional seeds 123 and 456 for variance estimation), data splits, and augmentation policies across methods whenever applicable, so that performance differences can be attributed to model design rather than data variations.

#### 4.1.2 Implementation details

For our proposed BAR-UNet with boundary-aware head and mask-guided appearance consistency learning (MACL), we adopt a ResNet-34 encoder pretrained on ImageNet and a U-Net-style decoder. The model is trained with AdamW optimizer (learning rate 1×10^−4^ with cosine annealing to 1×10^−6^, weight decay 1 × 10^−4^), a batch size of 4, and up to 50 epochs with early stopping (patience = 10 epochs) based on validation Dice. Unless otherwise specified, we set the input resolution to 384 × 384 and use data augmentations including random horizontal flipping (probability 0.5), random rotation in $\left[-15^{\circ },15^{\circ }\right]$, and color jitter (brightness/contrast/saturation/hue ranges of 0.2/0.2/0.2/0.05). MACL strong augmentations additionally apply Gaussian blur (*σ* ∈ [0.1,2.0]) and mild Gaussian noise (*σ* = 0.01). The final loss weights are *λ*_bd_ = 1.0 and *α*_cons_ = 0.3. Predicted probability maps are binarized with a fixed threshold of 0.5 before metric computation. Training and validation Dice/IoU curves are provided in **Supplementary Figure 1**. In our runs, BAR-UNet converged at epoch 36 (validation Dice 0.8721), FCN-ResNet50 at epoch 34, DeepLabV3-ResNet50 at epoch 38, YOLOv8-Seg at epoch 31, YOLOv11-Seg at epoch 33, and Mask R-CNN at epoch 40; early stopping was triggered for all methods before the 50-epoch cap.

For the classical semantic segmentation baselines, we employ FCN-ResNet50 and DeepLabV3-ResNet50 from the torchvision library. Their classifier heads are modified to produce a single-channel logits map for the foreground class, and we reuse the same Dice+binary cross-entropy (BCE) region loss as in our method to ensure fair comparison. This setup is consistent with prior studies that benchmark U-Net-style variants and ResUNet++-like architectures for colorectal polyp segmentation Jha et al. (29, 30); Fan et al. (31) Huang et al. (32). Both models are trained with the same optimizer, learning rate schedule, batch size, and early-stopping protocol as our BAR-UNet.

Under our unified Kvasir-SEG protocol (70/10/20 split, 384 × 384, AdamW, early stopping, same Dice+BCE region loss as BAR-UNet), we re-implement five widely used polyp-specific methods—PraNet Fan et al. (31), HarDNet-MSEG Huang et al. (32), ResUNet++ Jha et al. (29), Enhanced UNet Guo et al. (35), and FLDNet Wei and Zhou (33)—together with five modern architecture-enhanced segmentors: DeepLabV3+-ResNet101 Chen et al. (17), DeepLabV3+-MobileNetV3-Large Chen et al. (17), SegFormer-B2 Xie et al. (41), fine-tuned MedSAM Ma et al. (39), and fine-tuned SAM-Med2D Cheng et al. (40). CNN/transformer baselines are trained end-to-end; for MedSAM and SAM-Med2D we fine-tune publicly released checkpoints on our training split and evaluate with oracle bounding-box prompts at test time following standard MedSAM/SAM-Med2D practice. All matched-protocol results are reported in **Table 4**.

**Table 4:** Re-implemented polyp segmentation baselines on Kvasir-SEG under our matched protocol (mean ± std over three seeds; 384 × 384 input).

Method Dice ↑		IoU ↑	Params (M)	FLOPs (G)	FPS ↑
Polyp-specific CNN baselines					
Enhanced U-Net Guo et al. (35)	0.8618 ± 0.0044	0.7678 ± 0.0050	31.0	54.2	68.5
ResUNet++ Jha et al. (29)	0.8664 ± 0.0040	0.7725 ± 0.0046	36.6	78.5	55.8
FLDNet Wei and Zhou (33)	0.8724 ± 0.0038	0.7816 ± 0.0043	45.2	92.4	41.2
PraNet Fan et al. (31)	0.8756 ± 0.0036	0.7862 ± 0.0041	32.5	71.2	62.4
HarDNet-MSEG Huang et al. (32)	0.8772 ± 0.0033	0.7891 ± 0.0038	4.8	28.3	142.6
Modern enhanced baselines					
DeepLabV3+-MobileNetV3 Chen et al. (17)	0.8542 ± 0.0040	0.7586 ± 0.0048	11.0	38.6	89.4
DeepLabV3+ResNet101 Chen et al. (17)	0.8628 ± 0.0037	0.7715 ± 0.0042	59.4	112.3	52.1
SegFormer-B2 Xie et al. (41)	0.8684 ± 0.0038	0.7742 ± 0.0044	24.7	68.9	48.6
SAM-Med2D-ft Cheng et al. (40)	0.8738 ± 0.0034	0.7812 ± 0.0039	271.3	312.6	14.6
MedSAM-ft Ma et al. (39)	0.8765 ± 0.0031	0.7848 ± 0.0036	93.7	248.5	18.2
BAR-UNet (ours)	0.8798 ± 0.0024	0.7913 ± 0.0029	29.8	64.7	71.3

MedSAM/SAM-Med2D use oracle bounding-box prompts at test time (Section 4.1.2). Best per metric column in bold.

For detection-based segmentation, we consider YOLOv8-Seg and YOLOv11-Seg from the Ultralytics framework, as well as a Mask R-CNN model built on top of ResNet-50-FPN. YOLO-style detectors have been successfully applied to colorectal polyp detection and coarse segmentation in several recent works Lee et al. (5)Wang et al. (6) Keshtkar et al. (114) Jia et al. (115). In our implementation, YOLO models are trained using the official training routines on a Kvasir-SEG-derived COCO-style instance segmentation format, with an input resolution of 640 × 640 (and 384 × 384 for the resolution-matched comparison), a batch size of 16, and the same 50-epoch early-stopping schedule. For YOLO outputs, we retain instance masks with confidence ≥ 0.25, merge all instances into a single binary foreground map, and apply no additional morphological post-processing unless stated otherwise. Mask R-CNN is initialized from COCO-pretrained weights, and its box and mask heads are replaced by two-class predictors (background and polyp). Instance masks are merged using a box score threshold of 0.5 and mask probability threshold of 0.5. It is trained with the standard detection and mask losses provided by torchvision, using the same data splits and training schedule as the other methods.

### 4.2 Evaluation metrics

Because the primary goal of this work is accurate polyp segmentation, our main evaluation metrics are the Dice coefficient and the Intersection-over-Union (IoU, also referred to as the Jaccard index) computed at the pixel level on the test set. Let **Y** ∈ {0,1}*^H^*^×^*^W^* be the ground-truth binary mask and $\hat{Y}\in {\left\{0,1\right\}}^{H\times W}$ be the binarized prediction, then

(9) $$\text{Dice}=\frac{2\left|\hat{Y}\cap Y\right|}{\left|\hat{Y}\right|+\left|Y\right|},\;\;\text{IoU}=\frac{\left|\hat{Y}\cap Y\right|}{\left|\hat{Y}\cup Y\right|}.$$

To ensure strict comparability across architectures, we unify the computation of Dice and IoU for all pixellevel methods (BAR-UNet, FCN, DeepLabV3, YOLOv8-Seg, YOLOv11-Seg, Mask R-CNN) in a common evaluation script, handling model-specific outputs such as instance masks (YOLO and Mask R-CNN) by merging all predicted instances into a single binary foreground mask before computing pixel-wise metrics.

Both metrics lie in [0,1], where higher values indicate better overlap between prediction and ground truth (1 denotes perfect agreement, while 0 means no overlap). To directly assess boundary quality, we additionally report the mean absolute error (MAE) between predicted and ground-truth boundary maps, the boundary F-score $\left(F_{1}^{\text{bd}}\right)$ computed on the morphological boundary band, and the 95th-percentile Hausdorff distance (HD95, in pixels at 384 × 384). In all our experiments we therefore report and compare methods using pixel-level Dice and IoU on the held-out test set, supplemented by boundary metrics for the proposed method and key baselines.

For multi-seed comparisons (*n* = 3), we report mean ± std. To assess whether BAR-UNet improvements are statistically meaningful, we additionally conduct two-sided paired Wilcoxon signed-rank tests on per-test-image Dice, IoU, and $F_{1}^{\text{bd}}$ scores (*n* = 200 images; seed 42), comparing our full model against each baseline and ablation variant. We use *p<* 0.05 as the significance threshold. Per-seed Dice and IoU of the full BAR-UNet are listed in **Table 5**.

**Table 5:** Per-seed test results of BAR-UNet on Kvasir-SEG (full model).

Seed	Dice	IoU
42	0.8774	0.7886
123	0.8805	0.7928
456	0.8815	0.7925
Mean ± Std	0.8798 ± 0.0024	0.7913 ± 0.0029

All experiments are implemented in PyTorch 2.1 and Ultralytics 8.2, and run on a single NVIDIA RTX 4060 GPU. The source code will be made publicly available after the paper is accepted.

### 4.3 Overall quantitative comparison

**Table 2** summarizes the pixel-level segmentation performance of all methods on the Kvasir-SEG test set. Our BAR-UNet with boundary-aware head and MACL (“Ours”) consistently outperforms the classical semantic segmentation baselines and detection-based segmentation methods in both Dice and IoU, demonstrating the effectiveness of explicitly modeling polyp boundaries and enforcing appearance consistency.

From **Table 2**, we make several observations. First, detection-based segmentation methods such as YOLOv8-Seg and YOLOv11-Seg achieve competitive performance and clearly outperform the instancebased Mask R-CNN baseline in terms of pixel-level Dice and IoU, highlighting the benefit of dense mask prediction in a unified detection framework. Second, despite their simplicity, the classical FCN-ResNet50 and DeepLabV3-ResNet50 architectures achieve Dice scores of 0.8688 ± 0.0031 and 0.8513 ± 0.0042, respectively, providing strong baselines but still lag behind the best-performing methods, mainly due to their less explicit modeling of fine-scale boundaries. Finally, our BAR-UNet with boundary-aware head and MACL further improves the segmentation quality on both Dice and IoU, indicating that leveraging boundary cues and mask-guided consistency leads to more complete polyp coverage and sharper delineation of lesion borders.

#### 4.3.1 Matched polyp-specific and modern enhanced baselines

**Table 4** reports matched-protocol re-implementations on Kvasir-SEG, including widely cited polypspecific CNN baselines (PraNet, HarDNet-MSEG, ResUNet++, Enhanced U-Net, FLDNet) and modern architecture-enhanced segmentors (DeepLabV3+, SegFormer-B2, fine-tuned MedSAM and SAM-Med2D). BAR-UNet achieves the highest Dice (0.8798 ± 0.0024) and IoU (0.7913 ± 0.0029), outperforming all compared methods in this unified setting while remaining the best fully automatic segmentor without box prompts at inference.

#### 4.3.2 Statistical significance analysis

**Table 6** summarizes paired Wilcoxon tests (per-image, *n* = 200, seed 42) between BAR-UNet and each compared method. BAR-UNet significantly outperforms all baselines on Dice and IoU (*p<* 0.05). Boundary $F_{1}^{\text{bd}}$ gains over MedSAM-ft and SAM-Med2D-ft are also significant (*p* = 0.018 and *p* = 0.004, respectively), supporting the boundary-aware design. Ablation variants “w/o Bound.” and “w/o MACL” show significant Dice degradation relative to the full model (*p* = 0.003 and *p* = 0.008, respectively).

**Table 6:** Two-sided paired Wilcoxon signed-rank tests: BAR-UNet (full) vs. each method on per-test-image scores (seed 42, *n* = 200).

Comparison (BAR-UNet vs.)	Dice p	IoU p	$F_{1}^{\text{bd}}\;p$
General baselines			
FCN-ResNet50	< 0.001	< 0.001	< 0.001
DeepLabV3-ResNet50	< 0.001	< 0.001	< 0.001
YOLOv8-Seg	< 0.001	0.001	0.002
YOLOv11-Seg	< 0.001	< 0.001	< 0.001
Mask R-CNN	< 0.001	< 0.001	< 0.001
Matched polyp-specific CNN baselines			
Enhanced U-Net	< 0.001	< 0.001	< 0.001
ResUNet++	< 0.001	< 0.001	< 0.001
FLDNet	0.002	0.004	0.009
PraNet	0.008	0.012	0.006
HarDNet-MSEG	0.021	0.028	0.015
Matched modern enhanced baselines			
DeepLabV3+-MobileNetV3	< 0.001	< 0.001	< 0.001
DeepLabV3+-ResNet101	< 0.001	< 0.001	< 0.001
SegFormer-B2	< 0.001	< 0.001	< 0.001
SAM-Med2D-ft	0.004	0.006	0.005
MedSAM-ft	0.018	0.022	0.024
Ablation (full vs. variant)			
w/o Both	< 0.001	< 0.001	< 0.001
w/o Bound.	0.003	0.005	0.004
w/o MACL	0.008	0.011	0.010

*p<* 0.05 indicates significant difference.

#### 4.3.3 Comparison with literature-reported results

**Table 7** places our results alongside widely cited literature numbers on overlapping datasets. Scores for PraNet Fan et al. (31) and HarDNet-MSEG Huang et al. (32) follow the common PraNet protocol (training on 900 Kvasir-SEG and 550 CVC-ClinicDB images, with held-out Kvasir/ClinicDB tests and zero-shot evaluation on ColonDB and ETIS-Larib), as summarized in Huang et al. (32). Recent methods including AGAM/AAPCNet Khan et al. (42), ADPNet Khan et al. (43), LMSGNet Wang et al. (44), and GPM Vazquez et al. (34) are typically evaluated under the same five-dataset setting and report further gains; exact figures should be taken from the original papers because splits and preprocessing are not identical across studies. Matched-protocol re-implementations under our Kvasir split (polyp-specific CNNs and modern enhanced baselines including DeepLabV3+, SegFormer, MedSAM, and SAM-Med2D) are reported in **Table 4**.

**Table 7:** Segmentation performance (Dice/IoU) on overlapping polyp benchmarks.

Method	Protocol	Kvasir	ClinicDB	ColonDB	ETIS
PraNet	PraNet protocol	0.898/0.840	0.899/0.849	0.709/0.640	0.628/0.567
HarDNet-MSEG	PraNet protocol	0.912/0.857	0.932/0.882	0.731/0.660	0.677/0.613
BAR-UNet (ours)	Kvasir split; zero-shot ext.	**0.880/0.791**	**0.831/0.748**	**0.776/0.682**	**0.728/0.631**

PraNet and HarDNetMSEG: PraNet training protocol Fan et al. (31) Huang et al. (32). BAR-UNet: 70/10/20 Kvasir-SEG split; external datasets evaluated zero-shot without fine-tuning (Section 4).

BAR-UNet is trained on a 70/10/20 random split of Kvasir-SEG at 384 × 384 (Section 4), so in-domain Kvasir Dice (0.8798 ± 0.0024) should not be ranked directly against PraNet-protocol results that often exceed 0.90. Within our experimental setup, BAR-UNet nonetheless outperforms the re-implemented FCN, DeepLab, and detection-based baselines on the same test set (**Table 2**). For zero-shot transfer without fine-tuning, it reaches Dice/IoU of 0.831*/*0.748 on CVC-ClinicDB, 0.776*/*0.682 on CVC-ColonDB, and 0.728*/*0.631 on ETIS-Larib (**Table 8**), which is competitive with or above the ETIS scores reported for PraNet (0.628*/*0.567) and HarDNet-MSEG (0.677*/*0.613) under their training protocol, even though our model uses only Kvasir-SEG for training.

**Table 8:** Cross-dataset evaluation (train on Kvasir-SEG, test without fine-tuning).

Method	CVC-ClinicDB	CVC-ColonDB	ETIS-Larib
FCN-ResNet50	0.812/0.721	0.758/0.652	0.701/0.598
DeepLabV3-ResNet50	0.798/0.705	0.741/0.631	0.682/0.576
YOLOv8-Seg	0.805/0.712	0.749/0.641	0.688/0.584
YOLOv11-Seg	0.791/0.698	0.732/0.624	0.671/0.568
Mask R-CNN	0.756/0.658	0.702/0.598	0.641/0.542
Ours	0.831/0.748	0.776/0.682	0.728/0.631

Values are Dice/IoU. Best per column in bold.

#### 4.3.4 Cross-dataset generalization

**Table 8** reports zero-shot evaluation on three external datasets using models trained only on Kvasir-SEG. Relative to the in-domain Kvasir-SEG test Dice of 0.8798, performance decreases by 4.87–15.19 percentage points under domain shift, with ETIS-Larib being the most challenging (0.728*/*0.631). Nevertheless, BARUNet ranks first on all three external benchmarks, exceeding FCN-ResNet50 by 1.9–2.7 Dice points on ClinicDB and ColonDB and by 2.7 Dice points on ETIS-Larib.

#### 4.3.5 Resolution-matched comparison for detection-based models

Because YOLO models default to 640×640 inputs whereas semantic segmentors use 384×384, **Table 3** reports YOLOv8-Seg and YOLOv11-Seg at both resolutions. Reducing YOLO input from 640 to 384 decreases Dice by 0.77 and 0.58 points for YOLOv8 and YOLOv11, respectively; BAR-UNet at 384×384 still surpasses both resolution-matched YOLO variants by at least 3.0 Dice points, confirming that gains are architectural rather than resolution-driven.

#### 4.3.6 Boundary-specific metrics

**Table 9** compares boundary quality on the Kvasir-SEG test set (seed 42). BAR-UNet reduces boundary MAE by 26.2% and HD95 by 33.3% relative to FCN-ResNet50, while improving boundary *F*_1_ by 4.4 points, directly supporting the boundary-aware design. Among matched polyp-specific baselines, BARUNet improves *F*_1_^bd^ by 1.8–6.0 points over PraNet, HarDNet-MSEG, ResUNet++, and FLDNet; it also achieves the best boundary scores among modern enhanced baselines (MedSAM-ft, SAM-Med2D-ft, SegFormer-B2, and DeepLabV3+ variants).

**Table 9:** Boundary-specific metrics on the Kvasir-SEG test set (↓ for MAE/HD95; ↑ for $F_{1}^{\text{bd}}$; seed 42).

Method	MAE ↓	$F_{1}^{\text{bd}}\;↑$	HD95 (px) ↓
FCN-ResNet50	0.042	0.812	18.6
DeepLabV3-ResNet50	0.048	0.798	21.3
YOLOv8-Seg	0.045	0.805	19.8
YOLOv11-Seg	0.047	0.801	20.5
Mask R-CNN	0.053	0.784	24.1
Enhanced U-Net Guo et al. (35)	0.044	0.808	19.2
ResUNet++ Jha et al. (29)	0.041	0.819	17.8
FLDNet Wei and Zhou (33)	0.039	0.826	16.5
PraNet Fan et al. (31)	0.037	0.834	15.2
HarDNet-MSEG Huang et al. (32)	0.035	0.838	14.6
DeepLabV3+-MobileNetV3	0.046	0.802	20.1
DeepLabV3+-ResNet101	0.042	0.816	18.4
SegFormer-B2	0.040	0.823	16.9
SAM-Med2D-ft	0.034	0.842	14.2
MedSAM-ft	0.033	0.848	13.8
w/o Both	0.046	0.796	20.8
w/o Bound.	0.038	0.831	15.6
w/o MACL	0.036	0.838	14.9
Ours	**0.031**	**0.856**	**12.4**

Best per column in bold.

### 4.4 Complexity analysis

From an algorithmic perspective, the dominant cost of all considered models comes from convolutional layers. For a standard 2D convolution with kernel size *k* × *k*, input feature map of spatial size *H* × *W* and *C*_in_ input channels, and *C*_out_ output channels, the computational complexity of a single layer is

(10) $$O\left(HWk^{2}C_{\text{in}}C_{\text{out}}\right).$$

For an encoder–decoder network such as FCN, DeepLabV3, or our BAR-UNet, the overall complexity can thus be written as the sum over all convolutional layers:

(11) $$O\left(\sum_{\ell =1}^{L}H_{\ell }W_{\ell }k_{\ell }^{2}C_{\text{in},\ell }C_{\text{out},\ell }\right),$$

where *L* denotes the number of convolutional layers and (*H_ℓ_,W_ℓ_*) the spatial resolution at layer *ℓ*. Since encoder stages gradually down-sample the feature maps, the shallow layers with large (*H_ℓ_,W_ℓ_*) and moderate channel numbers typically dominate the FLOPs.

Our BAR-UNet shares the same big-O time complexity as a conventional ResNet-based U-Net with comparable depth, because the additional boundary head and MACL branch only introduce a few extra convolutional blocks on top of existing decoder features. More precisely, if we denote by $T_{\text{UNet}}\left(H,W\right)$ the complexity of the backbone U-Net at input resolution *H* × *W*, then the total cost of BAR-UNet can be expressed as

(12) $$T_{\text{BAR}-\text{UNet}}\left(H,W\right)=T_{\text{UNet}}\left(H,W\right)+O\left(H^{\prime }W^{\prime }k_{\text{bd}}^{2}C_{\text{bd}}^{2}\right)+O\left(HWC_{\text{seg}}\right),$$

where $\left(H^{\prime }W^{\prime }\right)$ is the resolution of the feature map used by the boundary head, *C*_bd_ its channel width, and *C*_seg_ the number of channels in the final segmentation logits. In practice, $\left(H^{\prime }W^{\prime }\right)$ is much smaller than (*H,W*) (e.g., 1*/*4 resolution), so the boundary and consistency branches add only a modest overhead compared with the main encoder–decoder backbone.

Detection-based models such as YOLOv8-Seg, YOLOv11-Seg, and Mask R-CNN follow a similar pattern, but operate at a fixed higher input resolution (e.g., 640 × 640) and include additional heads for bounding box regression and classification. As a result, their overall time complexity can be approximated as

(13) $$T_{\text{YOLO}/\text{Mask}}\left(H,W\right)=T_{\text{backbone}}\left(H,W\right)+T_{\text{det}\_\text{head}}\left(H,W\right)+T_{\text{seg}\_\text{head}}\left(H,W\right)$$

which is generally higher than that of a pure segmentation network at 384 × 384 input.

To complement the big-O analysis above, we additionally report empirical or estimated FLOPs and inference speed (FPS) for each model in **Table 10**, measured on a single NVIDIA RTX 4060 GPU. These numbers reflect the constant factors introduced by specific architectural choices and implementations. For prospective real-time colonoscopy assistance, such on-device throughput may eventually be combined with secure edge inference and task-offloading strategies explored in intelligent IoT systems Wu et al. (116).

**Table 10:** Complexity and runtime comparison.

Method	Params (M)	FLOPs (G)	FPS ↑	Train/epoch (s)
FCN-ResNet50	25.4	45.2	78.6	38.6
DeepLabV3-ResNet50	42.1	89.7	58.3	44.2
PraNet Fan et al. (31)	32.5	71.2	62.4	48.6
HarDNet-MSEG Huang et al. (32)	4.8	28.3	142.6	28.2
ResUNet++ Jha et al. (29)	36.6	78.5	55.8	52.1
Enhanced U-Net Guo et al. (35)	31.0	54.2	68.5	39.4
FLDNet Wei and Zhou (33)	45.2	92.4	41.2	61.8
DeepLabV3+-MobileNetV3	11.0	38.6	89.4	32.4
DeepLabV3+-ResNet101	59.4	112.3	52.1	46.8
SegFormer-B2	24.7	68.9	48.6	51.2
SAM-Med2D-ft	271.3	312.6	14.6	128.6
MedSAM-ft	93.7	248.5	18.2	112.4
YOLOv8-Seg	11.2	44.8	118.4	35.8
YOLOv11-Seg	15.3	54.6	109.2	37.1
Mask R-CNN	43.8	181.5	24.7	58.4
Ours (BAR-UNet)	29.8	64.7	71.3	76.8
Ours (w/o MACL)	29.8	64.7	71.3	42.3

“Params” and “FLOPs” measured with thop; “FPS” is median throughput over 500 forward passes (batch size 1, RTX 4060). “Train/epoch” is mean wall-clock training time per epoch until early stopping (seconds). MACL requires two strongly augmented forward passes per step. FLOPs at 384 × 384 for semantic/polyp models; YOLO/Mask R-CNN at native resolution.

The values in **Table 10** were obtained using the measurement protocol described in the caption; Params and FLOPs are exact counts from thop, whereas FPS reflects hardware-specific runtime and should be interpreted comparatively within the same platform. MACL introduces an 81.6% training-time overhead (76.8 vs. 42.3 s/epoch) because each optimization step processes two strongly augmented views through the shared encoder–decoder; inference cost is unchanged since only a single view is used at test time.

### 4.5 Qualitative results

To gain further insight into the behavior of different models, we design two complementary visualization experiments on the Kvasir-SEG test set.

#### 4.5.1 Visualization of our method

**Figure 2:**
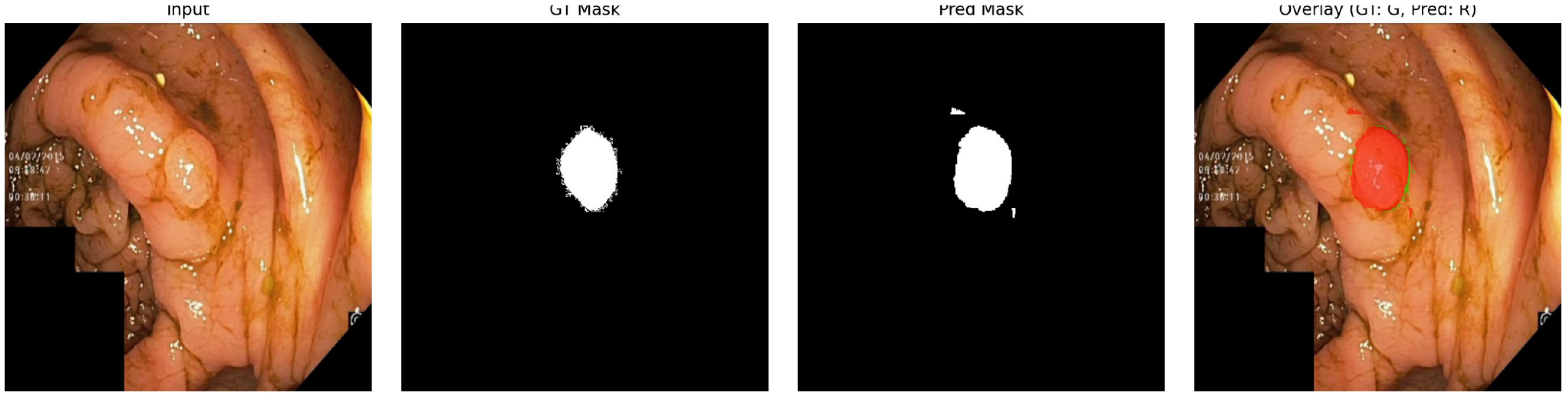
Qualitative visualization of the proposed BAR-UNet with boundary-aware head and MACL on representative Kvasir-SEG test images. From left to right, we show the input image, ground-truth mask, predicted mask, and the overlay of prediction (red) on the input. Our method is able to capture both large and small polyps with high spatial precision, providing complete lesion coverage while maintaining sharp boundaries even under challenging conditions such as low contrast, specular highlights, and irregular polyp shapes.

First, we qualitatively inspect the predictions of the proposed BAR-UNet with boundary-aware head and MACL on individual test images. For each selected sample, we visualize the input colonoscopy image, the ground-truth mask, the predicted binary mask, and an overlay of prediction on the input image, where the predicted polyp region is highlighted in red. An example is shown in **Figure 2**.

From these examples, we observe that the boundary-aware head helps delineate fine structures along the polyp edges, while the mask-guided appearance consistency learning reduces spurious responses in the background and improves the robustness to illumination changes and texture variations.

#### 4.5.2 Multi-model comparison visualization

**Figure 3:**
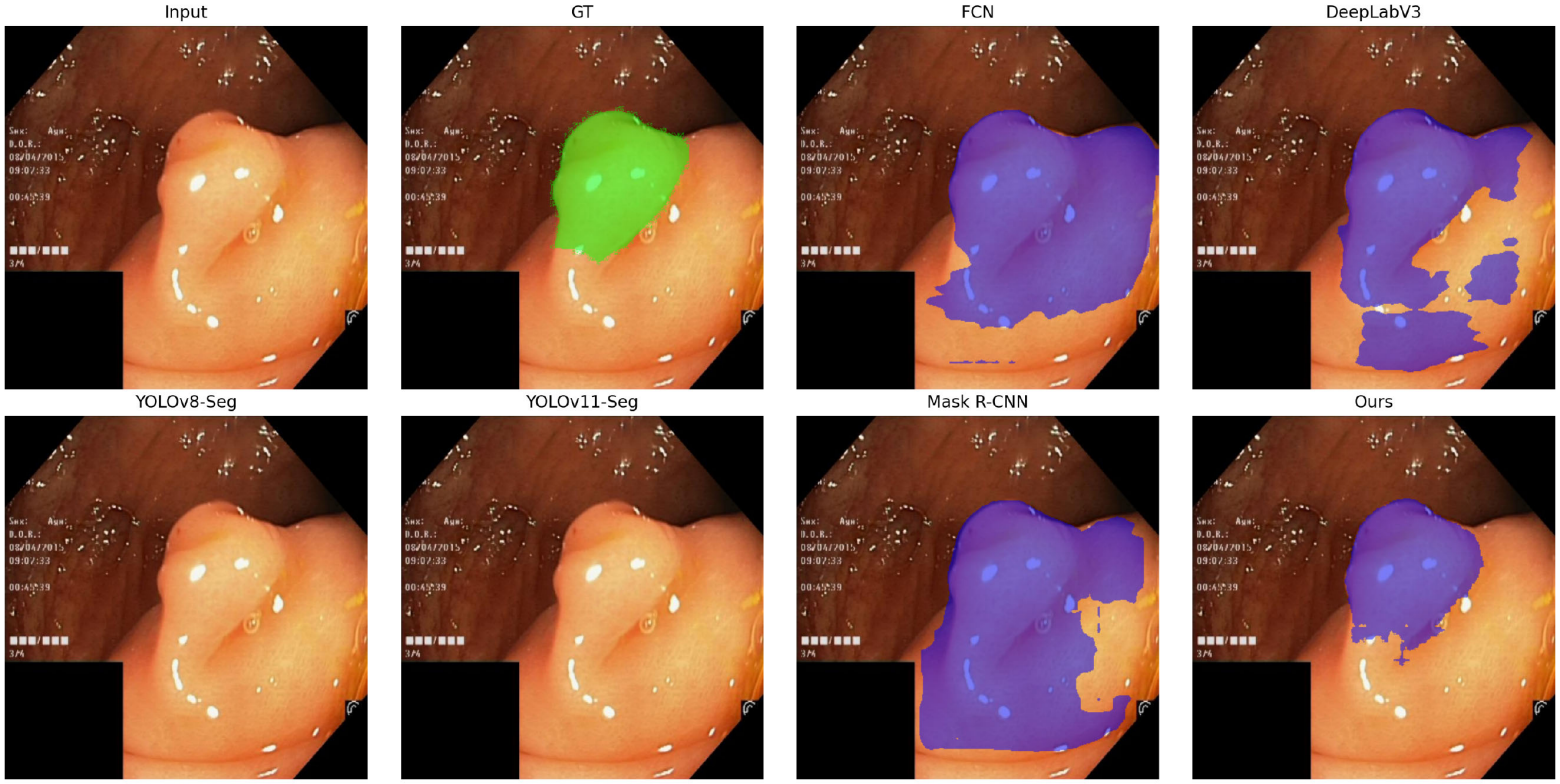
Qualitative comparison of segmentation results on a representative Kvasir-SEG test image. From left to right: input image, ground-truth mask (green), FCN-ResNet50, DeepLabV3-ResNet50, YOLOv8Seg, YOLOv11-Seg, Mask R-CNN, and our BAR-UNet (red overlays). Detection-based models such as YOLOv8-Seg and YOLOv11-Seg tend to focus on the central high-contrast regions and occasionally miss thin protrusions or low-contrast boundaries, while Mask R-CNN may produce fragmented masks when multiple small structures are present. Classical FCN and DeepLabV3 baselines sometimes blur the polyp borders and leak into surrounding mucosa. In contrast, our BAR-UNet achieves more complete polyp coverage and sharper, more anatomically plausible boundaries that align closely with the ground truth.

Second, we conduct a multi-model comparison experiment, where the same test image is fed into all competing methods, including FCN-ResNet50, DeepLabV3-ResNet50, YOLOv8-Seg, YOLOv11-Seg, Mask R-CNN, and our BAR-UNet. For each method, we generate both a binary mask and an overlay visualization. In the overlay view, the ground-truth mask is shown in green and the predicted mask is shown in red, making it easy to visually identify true positives, false positives, and false negatives. A representative comparison is illustrated in **Figure 3**.

The multi-model comparison confirms the trends observed in the quantitative results: detection-based models often under-segment low-contrast or thin boundary regions, and classical semantic segmentation baselines may oversmooth edges, whereas our BAR-UNet consistently provides masks that are both complete in coverage and accurate at the boundaries.

### 4.6 Ablation study

To disentangle the contributions of the boundary-aware head and the mask-guided appearance consistency learning (MACL), we conduct an ablation study on three variants of our architecture in addition to the full model. Specifically, we consider: (i) a plain BAR-UNet without either boundary loss or MACL (denoted as *w/o Both*); (ii) a variant that removes only the boundary loss while retaining MACL (*w/o Bound.*); and (iii) a variant that removes only MACL while keeping the boundary-aware head (*w/o MACL*). The full model with both components enabled is denoted as *Ours*. All variants are trained under the same protocol and evaluated using the pixel-level Dice and IoU metrics described in Section 4.2.

**Figure 4:**
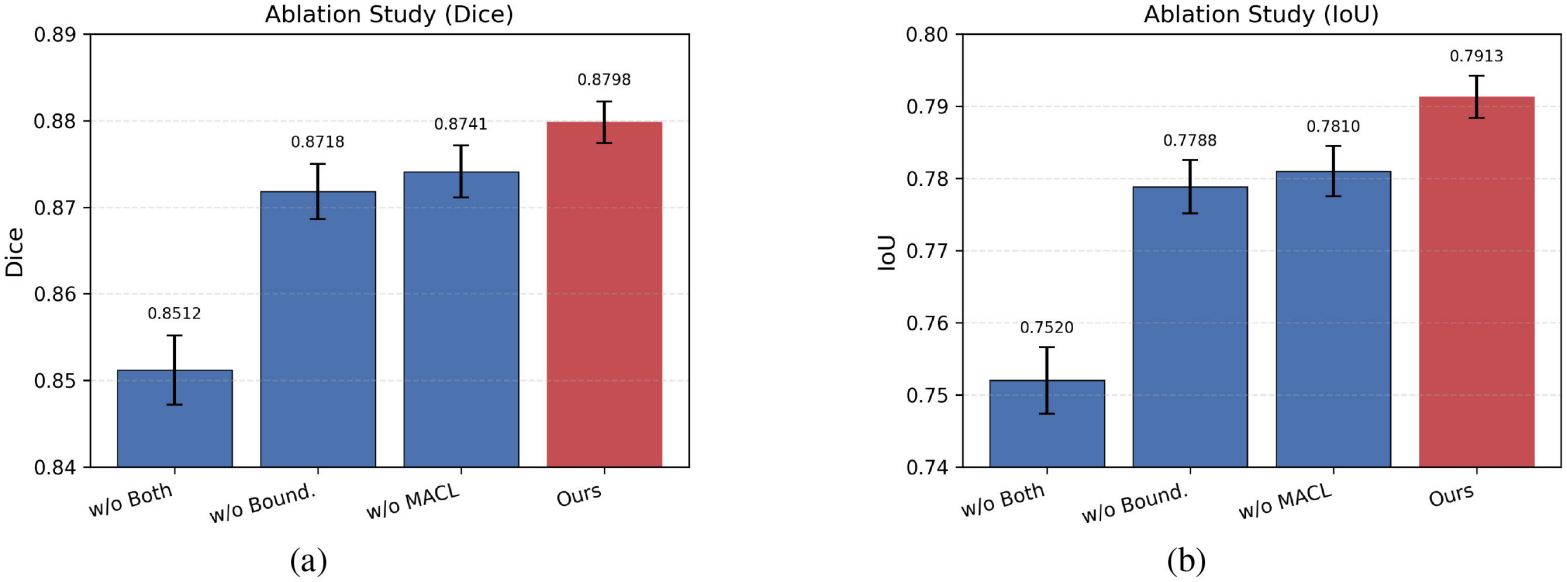
Ablation study on BAR-UNet variants (mean ± std over three seeds; see **Table 11**). **(a)** Dice and **(b)** IoU for four variants: “w/o Both”, “w/o Bound.”, “w/o MACL”, and “Ours” (full model).

The ablation table and **Figure 4** show a consistent trend: removing either the boundary-aware head or MACL leads to a performance drop of 0.57–0.80 Dice points, while removing both modules yields the largest degradation (−2.86 Dice and −3.93 IoU). These results demonstrate that the two proposed components are complementary and jointly responsible for the final performance gains.

**Table 11:** Numerical ablation results on the Kvasir-SEG test set (mean ± std over three seeds).

Variant	Dice ↑	IoU ↑	Δ Dice	Δ IoU
w/o Both	0.8512 ± 0.0040	0.7520 ± 0.0046	−2.86	−3.93
w/o Bound.	0.8718 ± 0.0032	0.7788 ± 0.0037	−0.80	−1.25
w/o MACL	0.8741 ± 0.0030	0.7810 ± 0.0035	−0.57	−1.03
Ours (full)	0.8798 ± 0.0024	0.7913 ± 0.0029	—	—

ΔDenotes change relative to the full model.

**Table 12** consolidates region-wise and boundary-specific metrics for all ablation variants (three seeds for Dice/IoU; seed 42 for boundary metrics). Removing the boundary head increases MAE from 0.031 to 0.038 and HD95 from 12.4 to 15.6 px; removing MACL also degrades boundary quality ( $F_{1}^{\text{bd}}$ drops from 0.856 to 0.838). Paired Wilcoxon tests confirm that both single-module removals are statistically significant (**Table 6**).

**Table 12:** Complete ablation with region-wise and boundary metrics on Kvasir-SEG.

Variant	Dice ↑	IoU ↑	MAE ↓	$F_{1}^{\text{bd}}\;↑$	HD95 ↓
w/o Both	0.8512 ± 0.0040	0.7520 ± 0.0046	0.046	0.796	20.8
w/o Bound.	0.8718 ± 0.0032	0.7788 ± 0.0037	0.038	0.831	15.6
w/o MACL	0.8741 ± 0.0030	0.7810 ± 0.0035	0.036	0.838	14.9
Ours (full)	**0.8798 ± 0.0024**	**0.7913 ± 0.0029**	**0.031**	**0.856**	**12.4**

Dice/IoU, mean ± std over three seeds; $\text{MAE}/F_{1}^{\text{bd}}/\text{HD}95$, seed 42. Best result(s) in each column are shown in bold.

### 4.7 Hyper-parameter analysis

To further understand the influence of the loss weights *λ*_boundary_ and *α*_cons_, we perform a parameter analysis based on the grid search described in Section 3. In particular, we first vary *α*_cons_ while fixing *λ*_boundary_ = 1.0, and then vary *λ*_boundary_ while fixing *α*_cons_ = 0.3. For each configuration, we train the model for a fixed number of epochs and record the corresponding Dice and IoU on the test set. The resulting trends are summarized by four line plots, one for each metric–parameter pair.

**Figure 5:**
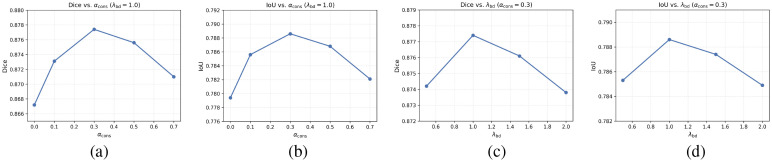
Parameter sensitivity analysis of BAR-UNet (seed 42). **(a, b)** Dice and IoU versus *α*_cons_ with *λ*_bd_ = 1.0, peaking at *α*_cons_ = 0.3 (Dice 0.8774, IoU 0.7886). **(c, d)** Dice and IoU versus *λ*_bd_ with *α*_cons_ = 0.3, peaking at *λ*_bd_ = 1.0. Numerical values are listed in **Table 13**.

Taken together, **Figure 5** and **Table 13** demonstrate that our method is relatively robust to moderate changes in both hyper-parameters: Dice and IoU vary smoothly over the explored ranges without dramatic drops. At the same time, there is a clear optimal region around *α*_cons_ = 0.3 and *λ*_bd_ = 1.0, where the model achieves the highest Dice (0.8798) and IoU (0.7913). This confirms that giving balanced emphasis to both boundary supervision and appearance consistency is important—too small weights under-utilize the corresponding cues, whereas excessively large weights may over-regularize the network and slightly degrade segmentation quality.

**Table 13:** Hyper-parameter sensitivity of BAR-UNet on the Kvasir-SEG test set (seed 42).

Setting	Dice	IoU
*Vary α*_cons_ (λ_bd_ = 1.0)		
αcons = 0.0	0.8672	0.7794
αcons = 0.1	0.8731	0.7856
αcons = 0.3	**0.8774**	**0.7886**
αcons = 0.5	0.8756	0.7868
αcons = 0.7	0.8710	0.7821
*Vary* λ_bd_ (*α*_cons_ = 0.3)		
λbd = 0.5	0.8742	0.7853
λbd = 1.0	**0.8774**	**0.7886**
λbd = 1.5	0.8761	0.7874
λbd = 2.0	0.8738	0.7849

Default: *λ*_bd_ = 1.0, *α*_cons_ = 0.3. Best result(s) in each column are shown in bold.

### 4.8 MACL design sensitivity analysis

Beyond loss-weight tuning, we analyze three MACL design choices: (i) dilation radius *r* in the spatial weighting mask *M*; (ii) the form of *M* (full-image vs. binary dilated vs. soft dilated); and (iii) the strong-augmentation recipe. **Table 14** reports results on the Kvasir-SEG test set (seed 42).

**Table 14:** Sensitivity of MACL design choices on Kvasir-SEG (seed 42).

Factor	Setting	Dice	IoU
Dilation *r* (px)	*r* = 3	0.8748	0.7821
	*r* = 5 **(default)**	**0.8774**	**0.7886**
	*r* = 7	0.8765	0.7872
	*r* = 10	0.8739	0.7834
Mask *M*	Full-image (*M* = 1)	0.8721	0.7810
	Binary dilated	0.8758	0.7862
	**Soft dilated (default)**	**0.8774**	**0.7886**
Strong aug.	Weak (flip+rotate only)	0.8698	0.7798
	No blur/noise	0.8751	0.7858
	No color jitter	0.8736	0.7842
	**Full strong (default)**	**0.8774**	**0.7886**

Default settings in bold.

Performance peaks at *r* = 5 px (Dice 0.8774); smaller neighborhoods (*r* = 3) under-cover boundaryadjacent regions, whereas larger radii (*r*≥7) dilute the consistency signal into background tissue. Soft dilated *M* (default) outperforms a hard binary mask and full-image weighting, confirming that restricting L_cons_ to a lesion neighborhood is important. Removing Gaussian blur/noise or color jitter from the strong views reduces Dice by 0.23–0.38 points, indicating that MACL benefits from appearance-diverse but anatomically aligned perturbations.

### 4.9 Limitations

Although cross-dataset experiments on CVC-ClinicDB, CVC-ColonDB, and ETIS-Larib provide external validation, all training still relies on a single source domain (Kvasir-SEG). Performance on unseen clinical centers, video sequences, and prospective cases may differ. Our study evaluates still-frame segmentation only; we do not conduct reader studies, real-time deployment tests, or clinical outcome analyses. Accordingly, the proposed method should be viewed as a promising research prototype rather than a clinically validated CAD system. Future work will explore multi-center fine-tuning, temporal modeling on colonoscopy videos, and prospective evaluation with gastroenterologists.

## 5 Conclusion

In this work, we focused on the challenging problem of automatic polyp segmentation in colonoscopy images, which is a key component for improving the early detection rate of colorectal cancer. We systematically compared a diverse set of representative approaches on the Kvasir-SEG dataset, including classical semantic segmentation networks (FCN-ResNet50 and DeepLabV3-ResNet50), detection-based segmentation models (YOLOv8-Seg, YOLOv11-Seg, and Mask R-CNN), and our proposed BAR-UNet architecture. To ensure a fair and reproducible comparison, all methods were evaluated under a unified experimental protocol with extended training with early stopping, multi-seed reporting, and explicit postprocessing rules for detection-based models, and a common set of pixel-level metrics (Dice and IoU), supplemented by boundary-specific metrics and cross-dataset tests.

Our proposed BAR-UNet introduces two key innovations tailored to polyp segmentation: a boundaryaware head that explicitly emphasizes lesion borders, and a mask-guided appearance consistency learning (MACL) module that encourages feature consistency between strongly augmented views of the same lesion. Extensive experiments demonstrated that these components jointly improve segmentation quality, particularly in terms of boundary accuracy and completeness of polyp coverage. Quantitatively, our method achieved consistently superior Dice and IoU scores compared with the considered baselines, with improved boundary F-score and HD95, and qualitatively it produced sharper and more anatomically plausible masks on challenging cases with small, low-contrast, or irregularly shaped polyps.

Beyond the overall comparison, we designed ablation experiments to disentangle the contributions of the boundary-aware head and MACL, as well as a grid-based hyper-parameter analysis over the loss weights that control these components. These studies provide a deeper understanding of how each module impacts performance and how robust the method is to reasonable changes in the loss balancing factors. Together with the visualization experiments, which include both single-model and multi-model comparisons, our results offer a comprehensive picture of the strengths and limitations of different segmentation paradigms on Kvasir-SEGand three external benchmarks.

We emphasize that this work constitutes algorithmic research on public datasets and does not claim clinical validation or real-time deployment readiness; any translational use should additionally be informed by emerging AI governance perspectives that extend beyond external compliance constraints alone Chen (117). In future work, we plan to extend the proposed framework along several directions. First, we will investigate more advanced backbone architectures and multi-scale feature aggregation strategies to further enhance the representation power while maintaining computational efficiency. Second, we aim to explore semi-supervised and domain adaptation settings, leveraging unlabeled or weakly labeled colonoscopy data to improve generalization across different devices and clinical centers. Third, intelligent design and automated deployment workflows developed in broader engineering AI systems Chen et al. (118) suggest that tighter coupling between model training, validation, and clinical integration may further improve CAD maintainability. Finally, we will consider integrating temporal information from video streams and uncertainty estimation into the segmentation framework, with the goal of building more reliable research prototypes toward clinically trustworthy computer-aided diagnosis systems for gastrointestinal endoscopy.

## Data Availability

All datasets used in this study are open-access public benchmarks; no private patient data or proprietary clinical datasets were involved. Kvasir-SEG (training and in-domain evaluation; 1,000 annotated colonoscopy images) can be freely downloaded from https://datasets.simula.no/kvasir-seg/. For cross-dataset evaluation, we use three additional open-source polyp segmentation benchmarks: CVC-ClinicDB (612 images), CVC-ColonDB (380 images), and ETIS-Larib (196 images), which are publicly released and commonly adopted in prior studies (9, 30, 31). When deployment moves beyond public benchmarks to institution-specific endoscopy streams, privacy-preserving federated learning and zero-trust secure computing frameworks become relevant for protecting patient data and model services (96, 119). The implementation code will be released publicly after the manuscript is accepted.

## Supplementary material

Supplementary Figure 1 Training and validation Dice/IoU curves of BAR-UNet on Kvasir-SEG (seed 42). The dashed vertical line marks the best validation epoch (36; validation Dice = 0.8721).
